# The Taxonomy and Phylogeny of *Echinometra* (Camarodonta: Echinometridae) from the Red Sea and Western Indian Ocean

**DOI:** 10.1371/journal.pone.0077374

**Published:** 2013-10-08

**Authors:** Omri Bronstein, Yossi Loya

**Affiliations:** Department of Zoology, The George S. Wise Faculty of Life Sciences, Tel-Aviv University, Tel-Aviv, Israel; University of Lausanne, Switzerland

## Abstract

The number of valid species in the genus *Echinometra* (Echinodermata, Echinoidea) and their associated identification keys have been debated in the scientific literature for more than 180 years. As the phylogeny and dispersal patterns of these species have been widely used as a prominent model for marine speciation, new insights into their taxonomy have the potential to deepen our understanding of marine speciation processes. In this study we examine *Echinometra* taxonomy, combining morphology and molecular tools. We present the taxonomy and phylogeny of Red Sea and Western Indian Ocean *Echinometra*. The currently available morphological keys were found to be limited in their ability to delineate all species within this genus. Nonetheless, morphological similarities between the Red Sea and Western Indian Ocean populations were high, and delimited them from the other species. These latter populations together formed a monophyletic clade, genetically distant from any of the other *Echinometra* species by more than 3%. Combining both traditional taxonomy and molecular evidence, we found that these populations were neither *Echinometra mathaei* nor *E. oblonga*, as previously considered. The morphological discrepancies of these populations, and their genetic divergence from the other *Echinometra* species, suggest that they should be considered as a new *Echinometra* species.

## Introduction

The genus *Echinometra* currently comprises eight species, two of them still undescribed, with species-level taxonomy of this genus yet to be completed [1–5]. *Echinometra* are of pan-tropical distribution and are often among the most prevalent urchins in the reefs they inhabit [2,6,7]. Though early studies of *Echinometra* suggested that only one species of this genus, *E. mathaei*, occurred in the Indo West Pacific (IWP) [8,9], later studies revealed the presence of four independent *Echinometra* species in that region [3,4,6,10–15]. These four closely-related IWP species occur sympatrically in Okinawa [6,11,14,16], and were originally referred to as *Echinometra* species A, B, C, and D [3]. Studies on both the morphological characteristics [3] and genetics [1,4] of these species, suggest that E. sp. B and *E*. sp. D in Okinawa are *E. mathaei* and *E. oblonga*, respectively; while E. sp. A and *E*. sp. C have remained unnamed. One of these four species, *E. mathaei*, has been called the world’s most abundant sea urchin [6], with distribution that spans from Hawaii and Tahiti throughout the Indo West Pacific (IWP), to the Western Indian Ocean (WIO) and the Red Sea (where it is the only *Echinometra* species reported) [2,6,17–19].

Though the taxonomy, phylogeny, and genetic structure of IWP *Echinometra* have been extensively studied [3,4,12–14,16], and much has been done in the eastern Pacific and tropical Atlantic [7,20,21], little has been done to date in regard to the Red Sea and WIO.

Here we investigated the taxonomy and phylogeny of Red Sea (Eilat) and WIO (Zanzibar) *Echinometra*. The four Okinawan *Echinometra* served as reference for delineating species from the other two regions. The phylogeny of *Echinometra* from the Red Sea and WIO is presented here for the first time. Our updated taxonomy of urchins from the latter two regions and their newly suggested phylogeny demonstrate the advantages of combining both molecular and morphological tools in delineating the boundaries and inferring relations between species of this genus.

## Materials and Methods

### Ethics statement

All field research and collection of specimens were approved by the local authorities in the country of collection, and permissions were granted as follows: Permit number 2007/28851, issued by the Israeli Nature and National Parks Protection Authority for collection in Eilat, Israel. Permit number AOl/VolXV/38, issued by the University of Dar Es Salaam, Institute of Marine Sciences for collection in Zanzibar, Tanzania. Sample collection in Okinawa was conducted as part of the 21^st^ Century Center of Excellence (COE) summer program, conducted at the University of the Ryukyus, Japan. Studies involving Okinawan *Echinometra* did not involve endangered or protected species and did not require a permit.

### Procedures and sample collection

**Figure 1:**
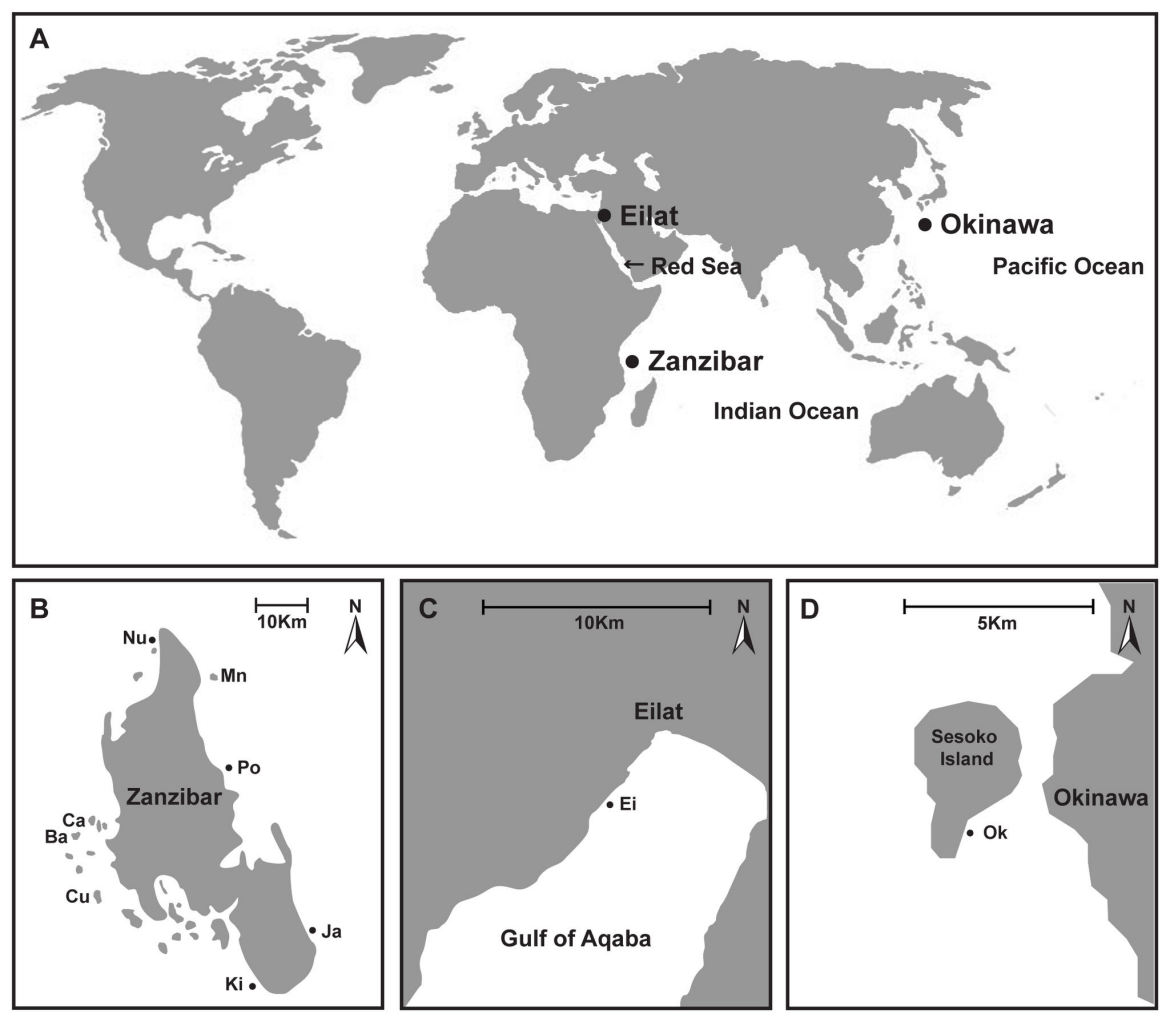
Collection sites of *Echinometra* specimens. (A) Map of the Indian Ocean and Eastern Pacific. Dots mark the three sampling sites: Okinawa, Zanzibar and Eilat. Detailed view of study sites: (B) Sesoko Isl., Okinawa, (C) Zanzibar, Western Indian Ocean, and (D) Eilat, Gulf of Aqaba/Eilat. Dots mark collection sites, scale bars indicate 1 km. Ca, Ba, Cu, Ki, Ja, Po, Mn, and Nu denote Changuu, Bawe, Chumbe, Kizimkazi, Jambiani, Pongwe, Mnemba and Nungwi, respectively. Ei and Ok denote Eilat and Okinawa, respectively.

Samples of *Echinometra* spp. were collected between June 2007 and November 2008 from three locations; Okinawa, Zanzibar, and Eilat (Gulf of Aqaba/Eilat, northern Red Sea) (Figure 1). A total of 69 individuals were collected from Zanzibar, 86 from Okinawa, and 42 from Eilat (Table 1). Samples were first morphologically identified using Mortensen’s criteria [8] and then sequenced and grouped based on the mtDNA phylogenetic tree reconstruction, in order to ratify the current taxonomical attributions. In this work Zanzibarian *Echinometra* are referred to as ZE and Eilat’s *Echinometra* as EE.

**Table 1:** Samples collection localities, GPS coordinates, depths and GenBank accession numbers of the sequences collected.

**Locality**	**Site**	**Coordinates**	**Depth (m)**	**Total number of samples**	**Number of sequenced samples**	**GenBank accession numbers**
IWP- Okinawa	Sesoko	26°38’03.44”N, 127°51’51.24”E	1	86	78	KC464982-KC465059
WIO-Zanzibar	Changu	06°07’03.10”S, 39°10’06.89”E	1-3	9	9	KC464904; KC464913; KC464937; KC464957; KC464960; KC464966; KC464975; KC464978-KC464979
	Bawe	06°08’16.36”S, 39°07’57.82”E	1-3	10	10	KC464885; KC464900; KC464912; KC464921; KC464934; KC464962; KC464964; KC464968; KC464974; KC464981
	Chumbe	06°16’54.51”S, 39°10’32.85”E	1	9	9	KC464899; KC464914-KC464916; KC464922; KC464936; KC464943; KC464963; KC464969
	Kizimkazi	06°28’12.81”S, 39°29’06.70”E	1	9	9	KC464918; KC464923-KC464925; KC464935; KC464954; KC464956; KC464959; KC464972
	Jambiani	06°19’57.32”S, 39°33’32.16”E	1-2	8	8	KC464901; KC464905-KC464906; KC464917; KC464958; KC464961; KC464967; KC464973
	Pongwe	06°01’20.99”S, 39°25’30.21”E	1-3	9	9	KC464888; KC464895; KC464930; KC464933; KC464940; KC464955; KC464971; KC464976-KC464977
	Mnemba	05°48’51.17”S, 39°21’33.47”E	1-2	9	9	KC464886-KC464887; KC464894; KC464907; KC464926; KC464938; KC464965; KC464970; KC464980
	Nungwi	05°43’47.44”S, 39°17’27.75”E	1-3	6	6	KC464902; KC464919; KC464927-KC464929; KC464939
RS- Gulf of Aqaba/Eilat	Eilat	29°30’59.10”N, 34°55’34.63”E	1-2	42	34	KC464879-KC464884; KC464889-KC464893; KC464896-KC464898; KC464903; KC464908-KC464911; KC464920; KC464931-KC464932; KC464941-KC464942; KC464944-KC464953

IWP: Indo West Pacific; WIO: Western Indian Ocean; RS: Red Sea

### Morphological measurements

The morphological array of characters used to delineate the species of *Echinometra* comprised: length, width and height of test, color of spines, milled rings (A small flange near the base of the spine marking the distal most limit of muscle attachment onto the spine base), and skin around the peristome, shape of spicules in the tube feet and gonads, the number of pore-pairs, and sperm morphology. The length and width of the tests were measured at the ambitus while height was measured along the oral-aboral axis. Measurements to the nearest 0.5 mm were performed using thin blade Vernier calipers to prevent interference by the spines. Color of the spines was described from live samples. Milled rings were determined as bright, faded, or dark, and the skin around the peristome as bright or dark. Following external examination the urchins were dissected and the gonads removed. Gonad samples were then used for spicule analysis, sex determination, and DNA extraction. In order to obtain tube feet spicules, several tube feet were detached with forceps, while gonad spicules were obtained from a small portion of the gonad. Tube feet and gonad spicule samples were photographed under X20 and X10 magnifications, respectively, using a light microscope, and analyzed using the software ImageJ [22]. The number of pore-pairs on every ambulacral plate was counted under a dissecting microscope. Pair-wise Kolmogorov-Smirnov tests were performed for every pair of samples to check for similarities in pore-pairs distribution. P-values were adjusted for multiple testing to minimize false-discovery-rate using the Bonferroni correction.

Examination of sperm was carried out using a scanning electron microscope (JEOL JSM5610LV). Sperm samples were obtained as undiluted semen, i.e., “dry sperm”, by removing the testes. The samples were then fixed in 2.5% glutaraldehyde in filtered (0.2 µm) seawater (FSW) and stored at 4°C. Further preparation of the samples followed a procedure modified from [3].

### DNA extraction, amplification and sequencing

DNA was extracted from the gonads based on a protocol following Pochon et al [23]. Following extraction, DNA concentrations were assessed using NanoDrop^®^ ND-1000 spectrophotometer. Extractions revealed as impure were discarded and the extraction was repeated. Otherwise, DNA was diluted to ca. 2.5 ng/µl with ddH_2_O. A ~600 bp long portion of the mitochondrial cytochrome c oxidase subunit Ι (COΙ) gene was amplified using the primers COΙ-f (5’-CCTGCAGGAGGAGGAGAYCC-3’) and COΙ-d (5’-GAACATGATGAAGAAGTGCACCTTCCC-3’ [24], which correspond to positions 6,439-7,039 in the *Strongylocentrotus purpuratus* mitochondrial genome. The polymerase chain reaction (PCR) was performed in 40 µl total volume (4 µl 10X reaction buffer, 3.2 µl dNTP (2.5 mM), 2.4 µl MgCl (25 mM), 0.8 µl (8 pmol) of each primer, 0.4 µl of GoTaq^®^ Flexi DNA Polymerase (Promega), 26.4 µl ddH_2_O and 2 µl of DNA template (ca. 5 ng/µl)). Amplifications were conducted in a Peqlab Primus 96 machine and used a standard amplification profile of an initial denaturation step at 94°C for 4 min followed by 35 cycles of 94°C for 30 sec, 55°C for 30 sec and 72°C for 30 sec and finished with 5 min at 72°C [24]. PCR products were purified using a Montage^®^ Millipore column kit and sequenced in forward and backward directions (Big-Dye v3.1, Applied Biosystems Inc., Foster City, CA, USA). The samples were then analyzed using an ABI 3730xl Genetic Analyzer (Applied Biosystems).

### Alignment and analysis of sequences

Chromatograms were checked manually using ChromasPro v1.42 (Technelysium Pty Ltd) and aligned using MAFFT v6 [25]. Eight additional *Echinometra* COΙ sequences were obtained from GenBank (accession numbers: AY262861, AY262940, AY262932, AY262886, AY262906; AF255471, AF255530 and AF255526, from Landry et al. [4] and McCartney et al. [7], respectively). *Heliocidaris crassispina* (Echinodermata, Echinoidea) (GenBank accession number: JN716400) was used as outgroup. New sequences were deposited in GenBank (accession numbers: KC464879 – KC465059). Phylogenetic reconstructions were performed using both Maximum Likelihood (ML) and Bayesian inference (BI) analyses. ML analysis was performed using raxmlGUI v1.1 [26] using the GTRGAMMA model, and Bayesian analysis was conducted using MrBayes v3.1.2 [27] with the generalized time-reversible model GTR+I+G, which was selected based on results from jModelTest [28,29]. RaxML tree reconstructions were carried out using 100 random starting trees. Branch support was composed based on 1,000 bootstrap replications. In the MrBayes analysis, two runs with four chains each were conducted, with default temperatures and prior distributions. The chains were sampled every 100 generations. Convergence was achieved at 3,000,000 generations. After convergence, the sampling continued until the analysis reached 20,000,000 generations. The first 3,000,000 generations, amounting to 15% of the total number of generations, were discarded as burnin. Genetic divergences within and among taxa were calculated as Kimura-2-parameter (K2P) distance [30] using MEGA v5.0 [31].

## Results

### Morphologically-based identification of *Echinometra* from Okinawa, Zanzibar, and Eilat

Summary of the morphological set of characters: color of spines, milled rings, skin of peristome, tube feet, and gonad spicules yielded 27 character-states arranged in 60 different character state combinations and summarized in Table 2. Based on these morphological criteria, individuals from Okinawa were assigned to four groups, corresponding to the four known IWP *Echinometra*; while individuals from Eilat and Zanzibar seemed to differ from these species based on the same characters. However, even among the IWP species, the current morphological keys were sometimes insufficient for conclusive species identification. For example, while E. sp. A is distinctively characterized by having white-tipped spines (found in 100% of the samples), the great diversity of spine color in E. sp. C (from light brown to violet, green, and black), rendered this character uninformative for this species. The unique white-tipped appearance of E. sp. A was never observed in ZE or EE. While the majority of ZE individuals (62.7%) resembled *E. oblonga* in bearing black spines, the rest of the population included character states shared by both *E. mathaei* and *E*. sp. C (i.e. light brown) or a new unique violet color. EE could not be discriminated from either *E. mathaei* or *E*. sp. C based on this character. Milled rings presented three character-states: ‘bright’, ‘dark’, and ‘faded’. While the first two appeared in individuals from all locations, the ‘faded’ morph was strictly confined to the Zanzibar and Eilat individuals (32.5% and 30%, respectively). The skin around the peristome was described as being either ‘dark’ or ‘bright’. These two character-states were not evenly distributed, as the ‘dark’ morph predominated in all but one species (*E*. sp. C). ZE and EE had highly similar proportions of peristomal skin coloration. For both, proportions of the bright-skinned individuals were very low (1.9% and 2.5%, respectively) in comparison to the dark-skinned individuals (98.0% and 97.5%, respectively). These proportions were close to those displayed by *E. oblonga* (entirely dark-skinned around the peristome), and furthest from *E. mathaei* and *E*. sp. A (62.2% and 75%, respectively).

**Table 2:** Morphological characteristics of *Echinometra* from Okinawa, Zanzibar and Eilat.

					**Dominant spicule types**		
**Locality**	**Species**	**Color of spines**	**Milled rings**	**Skin of peristome**	**Tube feet**	**Gonads**	**Ratio (%)**
Okinawa	*E*. sp. A	Various colors with white tip	Bright	Dark	Bihamate	Needle	75
	(n = 4)	Various colors with white tip	Bright	Bright	Bihamate	Needle	25
	*E. mathaei*	Light brown	Bright	Bright	Triradiate	Triradiate	2.22
	(n = 45)	Light brown	Dark	Bright	Bihamate	Needle	2.22
		Light brown	Dark	Bright	Bihamate	Triradiate	2.22
		Light brown	Dark	Bright	Needle	Triradiate	2.22
		Light brown	Dark	Bright	None^a^	Triradiate	2.22
		Light brown	Dark	Bright	None	None	2.22
		Light brown	Dark	Bright	Triradiate	Triradiate	15.56
		Light brown	Dark	Bright	Triradiate	None	4.44
		Light brown	Dark	Dark	Bihamate and triradiate	Needle	4.44
		Light brown	Dark	Dark	Bihamate	Needle	22.22
		Light brown	Dark	Dark	Bihamate	Triradiate	2.22
		Light brown	Dark	Dark	Triradiate	Needle	2.22
		Light brown	Dark	Dark	Triradiate	Triradiate	13.33
		Light brown	Dark	Dark	None	Triradiate	4.44
		Light brown	Dark	Dark	None	Triradiate and needle	2.22
		Dark brown	Dark	Dark	Bihamate and triradiate	Needle	4.44
		Dark brown	Dark	Dark	Triradiate	Triradiate and needle	2.22
		Dark brown	Dark	Dark	Triradiate	Triradiate	4.44
		Dark brown	Dark	Bright	Triradiate	Triradiate	4.44
	*E*. sp. C	Various colors	Bright	Bright	Triradiate	Triradiate	29.63
	(n = 27)	Various colors	Bright	Bright	Bihamate	Triradiate	3.70
		Various colors	Bright	Bright	None	Triradiate	7.41
		Various colors	Bright	Bright	Triradiate	Bihamate and triradiate	3.70
		Various colors	Bright	Bright	Triradiate	Bihamate	3.70
		Various colors	Bright	Bright	Triradiate	Needle	3.70
		Various colors	Bright	Bright	Triradiate	Triradiate and needle	3.70
		Various colors	Bright	Bright	Bihamate and triradiate	Triradiate and needle	3.70
		Various colors	Bright	Bright	Triradiate	NG^b^	3.70
		Various colors	Bright	Dark	Triradiate	Triradiate	22.22
		Various colors	Bright	Dark	Triradiate	Multiple^c^	3.70
		Various colors	Bright	Dark	None	None	3.70
		Various colors	Dark	Dark	Triradiate	Triradiate	3.70
		Various colors	Dark	Dark	Triradiate	None	3.70
	*E. oblonga*	Black	Dark	Dark	None	Triradiate and needle	40.0
	(n = 10)	Black	Dark	Dark	Triradiate	Triradiate and needle	10.0
		Black	Dark	Dark	Triradiate	Triradiate	10.0
		Black	Dark	Dark	Triradiate	Bihamate	10.0
		Black	Dark	Dark	None	Bihamate	10.0
		Black	Dark	Dark	Triradiate	Multiple	20.0
Zanzibar	ZE	Black	Dark	Dark	Bihamate	Needle	31.37
	(n = 69)	Black	Dark	Dark	Bihamate	Needle and bihamate	11.76
		Black	Dark	Dark	Bihamate	Needlle and 8^d^	19.61
		Light brown	Faded	Dark	Bihamate	Needle	19.61
		Light brown	Faded	Dark	Bihamate	Needlle and 8	3.92
		Violet	Faded	Dark	Bihamate	Needle	7.84
		Violet	Faded	Dark	Bihamate	Needlle and 8	3.92
		Violet	Bright	Bright	Bihamate	Needle	1.96
Eilat	EE	Light brown	Bright	Dark	Bihamate	Needle	40
	(n = 42)	Light brown	Bright	Dark	Bihamate	Needlle and 8	5
		Light brown	Bright	Dark	Bihamate	Multiple	2.5
		Dark brown	Faded	Dark	Bihamate	Needlle and 8	12.5
		Dark brown	Faded	Dark	Bihamate	Needle	5
		Dark brown	Faded	Dark	Bihamate	Multiple	5
		Light brown-green	Bright	Dark	Bihamate	Needle	15
		Light brown-green	Bright	Bright	Bihamate	Needlle and 8	2.5
		Dark brown-green	Faded	Dark	Bihamate	Needle	7.5
		Dark brown-green	Bright	Dark	Bihamate	Needle	2.5
		Dark brown-green	Bright	Dark	Bihamate	Multiple	2.5

External features and spicule assemblages in the tube feet and gonads of *Echinometra* from Okinawa, Zanzibar and Eilat. Ratio refers to the percentage of individuals sharing a specific set of character combination. Figures in parentheses indicate the number of individuals sampled. ^a^ None – no spicules were found; ^b^ NG – no gametes; ^c^ Multiple – combination of three or more types of spicules: bihamate, ‘figure-eight’ shaped, needle and triradiate; ^d^ 8 – 'figure-eight' shaped spicules.

**Figure 2:**
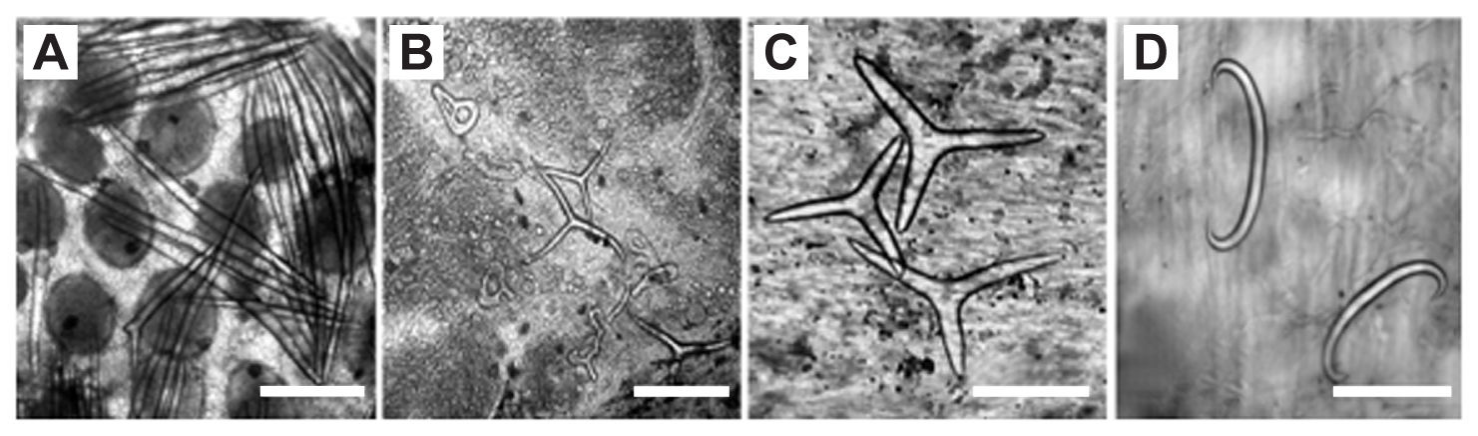
Spicule types of *Echinometra*. (A) Needle spicules in gonads of *E*. sp. EE. (B) Triradiate and 'figure-eight' shaped spicules in gonads of *E*. sp. C. (C) Triradiate spicules in tube feet of *E. oblonga*. (D) Bihamate spicules in tube feet of EE. Scale bars indicate 100 µm.

Four dominant spicule types (Figure 2) were encountered in the gonads and tube feet, arranged in seven different spicule combinations. Two additional spicule arrangements were defined as ‘none’ (i.e. having no spicules) and ‘multiple’ (i.e having a mixed array of spicules), bringing the total number of character-states of this character to nine. The tube feet spicules of E. sp. A, ZE, and EE were composed entirely of the bihamate type; all other species contained varying amounts of the triradiate spicule type. *E. mathaei* presented the greatest variety of tube feet spicules, with five different spicule categories, followed by E. sp. C with four different tube-feet spicule arrangements. In *E. oblonga* half of the population exhibited triradiate spicules while the other half had none. Gonad spicules were the most variable and diverse of all characters studied. This diversity is evident in the nine different character-states described from the studied populations. E. sp. A presented the most homogenous spicule array, with all spicules belonging to the needle type. In contrast, E. sp. C was the most diverse of all species studied, displaying seven different character states (Table 2). *E*. *mathaei* and *E. oblonga* had an intermediate level of diversity in this character, presenting four character-states each. The gonad spicules of ZE and EE were similar in composition and bore the highest resemblance to E. sp. A. In both ZE and EE the solely needle spicule type was the dominant character-state (60.7% and 70%, respectively); while the other two character-states of each of these species comprised needle spicules in various combinations with other spicule types.

### Number of pore pairs

**Figure 3:**
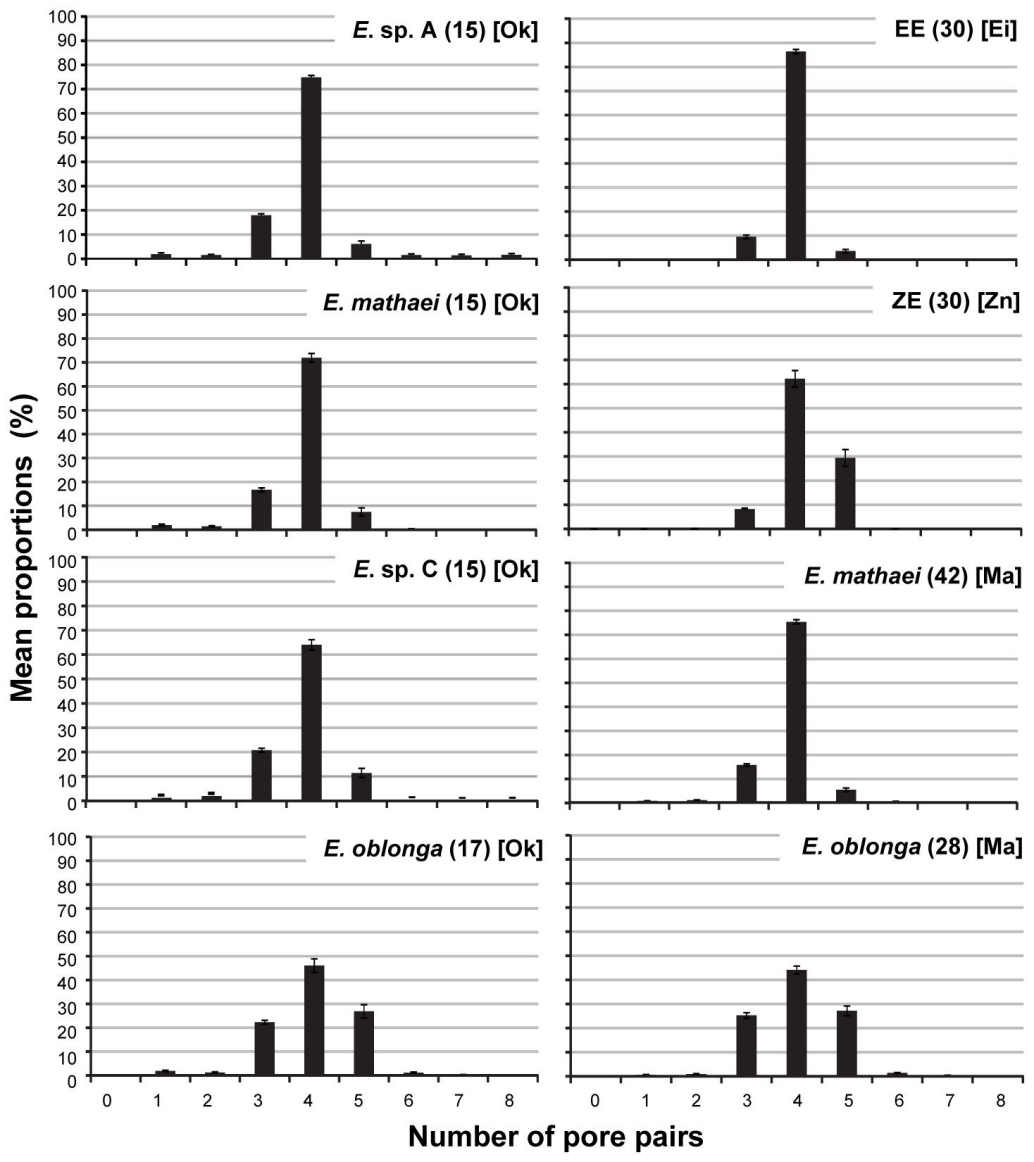
Pore-pairs ratios of *Echinometra* from Eilat, Zanzibar, Okinawa and Mauritius. Mean proportion ± SE (%) of pore-pairs in *Echinometra* species from Eilat [Ei], Zanzibar [Zn], Okinawa [Ok], and Mauritius [Ma]. Figures in parentheses represent sample sizes. Raw data on Okinawan and Mauritian *Echinometra* courtesy of Yuji Arakaki.

Pore-pairs ratios differed significantly between some species while in other species these ratios were statistically insignificant (Figure 3). Pore-pairs distribution in EE differed significantly from ZE (K-S test, D = 0.2534, P* < 0.01), Okinawan *E. oblonga* (K-S test, D = 0.2507, P* < 0.01), and Mauritian *E. oblonga* (K-S test, D = 0.2562, P* < 0.01) (Table 3). No significant differences were observed between EE and the other *Echinometra* studied. ZE was significantly different from *E. mathaei* of both Okinawan (K-S test, D = 0.2096, P* < 0.05) and Mauritian (K-S test, D = 0.231, P* < 0.01) origin. Nonetheless, apart from EE and ZE, statistically significant, Bonferroni corrected differences in pore-pairs distribution were only detected between *E. mathaei* and *E. oblonga* and not among any of the other *Echinometra* species (Table 3). * Bonferroni corrected P values.

**Table 3:** Pair wise comparisons of the number of pore-pairs between species of *Echinometra*.

	**EE**	**ZE**	**A [Ok]**	**B [Ok]**	**C [Ok]**	**D [Ok]**	**B [Ma]**	**D [Ma]**
**EE** (n = 30)		**	-	-	-	**	-	**
**ZE** (n = 30)	D = 0.2534 ***		-	*	-	-	**	-
**A [Ok**] (n = 15)	D = 0.1125 -	D = 0.1865 **		-	-	-	-	-
**B [Ok**] (n = 15)	D = 0.1061 -	D = 0.2096 ***	D = 0.0437 -		-	*	-	*
**C [Ok**] (n = 15)	D = 0.1467 -	D = 0.1694 *	D = 0.0434 -	D = 0.0406 -		-	-	-
**D [Ok**] (n = 17)	D = 0.2507 ***	D = 0.1719 **	D = 0.1837 **	D = 0.2068 ***	D = 0.1666 **		**	-
**B [Ma**] (n = 42)	D = 0.0923 -	D = 0.231 ***	D = 0.0445 -	D = 0.0214 -	D = 0.0616 -	D = 0.2282 ***		**
**D [Ma**] (n = 28)	D = 0.2562 ***	D = 0.1827 **	D = 0.1892 **	D = 0.2123 ***	D = 0.1721 *	D = 0.0165 -	D = 0.2337 ***	

Pair wise Kolmogorov-Smirnov comparisons of the number of pore pairs between *Echinometra* from Eilat, Zanzibar, Okinawa and Mauritius. EE - Eilat *Echinometra*, ZE – Zanzibar *Echinometra*, A [Ok] – Okinawan E. sp. A, B [Ok] – Okinawan *E. mathaei*, C [Ok] – Okinawan E. sp. C, D [Ok] – Okinawan *E. oblonga*, B [Ma] – Mauritian *E. mathaei*, and D [Ma] – Mauritian *E. oblonga*. K-S test statistics and uncorrected P values are given (below diagonal) as well as Bonferroni corrected P values (above diagonal) for each pair wise comparison. The symbols, ***, **, *, and -, indicate significant levels, P < 0.001, P < 0.01, P < 0.05, and non significant, respectively. Figures in parentheses indicate samples size.

### Sperm head morphology

**Figure 4:**
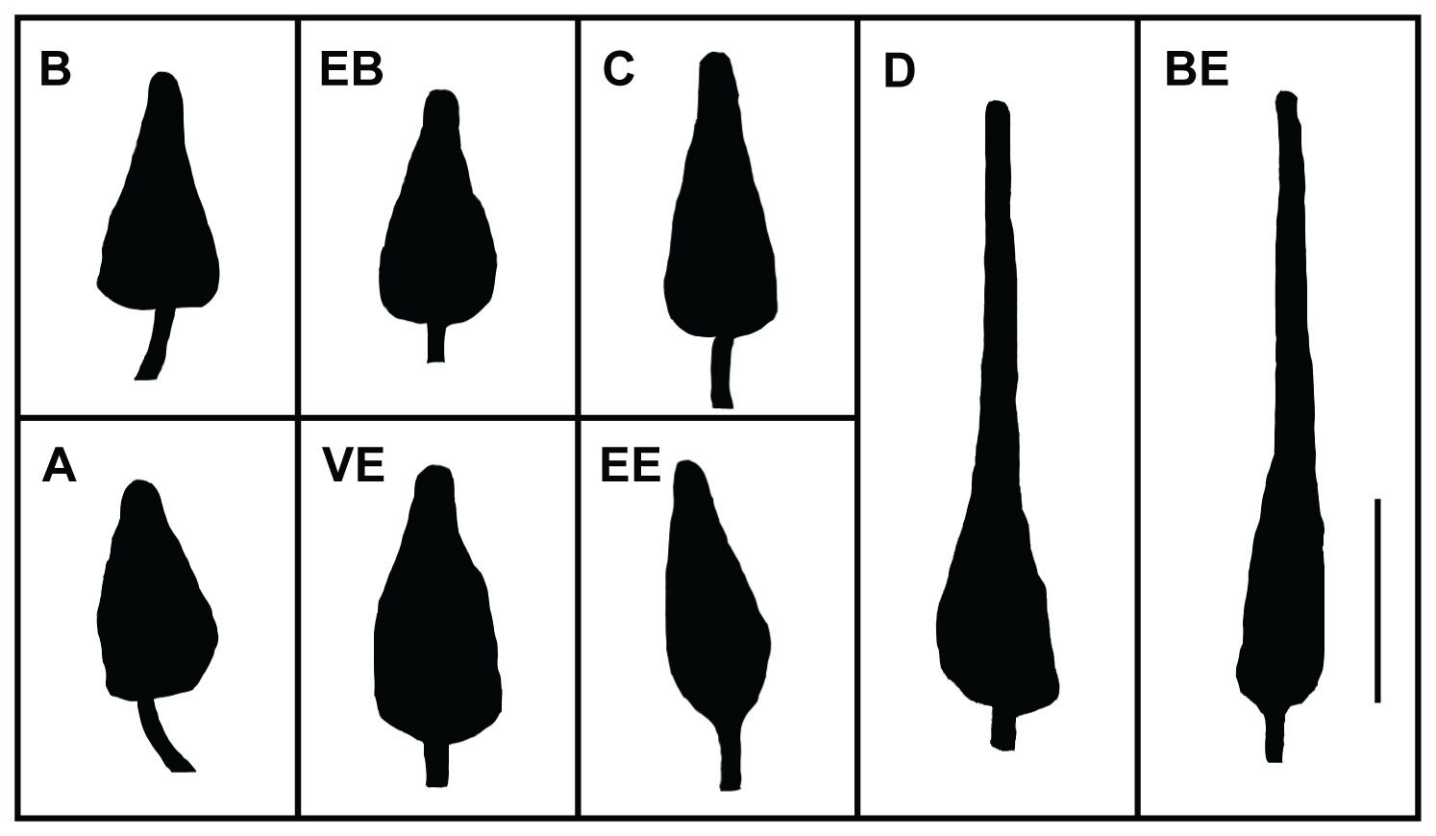
Sperm morphologies of Red Sea, Western Indian Ocean and Indo-West Pacific *Echinometra*.

The morphology of sperm heads varies greatly among species of *Echinometra*. In general, two types of sperm head morphologies are noted: compact, with a length over width ratio of about three to one; and elongated, with a length over width ratio of about six to one. At the time of observation, ZE sperm samples were found to be severely degraded, possibly due to inadequate preservation or exposure to excessive heat, consequently, data from these samples could not be trusted and was removed from further analyses. EE displayed the compact sperm morphology with an average length of 3.90 ± 0.34 µm and an average width of 1.27 ± 0.08 µm. The shape (i.e. proportion of length to width) averaged at 3.08 ± 0.29 µm. In that sense EE resembles E. sp. C and *E. mathaei*, yet significantly differs (student t-test, p < 0.05) from E. sp. A and *E. oblonga* (Figure 4, Table 4).

 Sperm head schematics of Okinawan (A, B, C, D), Mauritian (VE, EB, BE), and Eilat’s *Echinometra*. A, B, C, D, VE, EB, BE, and EE denote *Echinometra* sp. A, *E. mathaei*, *E*. sp. C, *E. oblonga*, Violet *Echinometra*, *E. mathaei*, *E. oblonga*, and Eilat *Echinometra*, respectively. The bar indicates 2 µm. Sperm head illustrations of Okinawan and Mauritian Echinometra were modified after Arakaki et al. (1998).

**Table 4:** Sperm head allometrics of *Echinometra*.

**Locality**	**Species**	**# of sperm sampled**	**Length (µm)**	**Width (µm)**	**Ratio (Length/Width)**	**Source**
Okinawa	*E*. sp. A	50	2.91 ± 0.19	1.24 ± 0.05	2.34 ± 0.18	Arakaki et al. 1998
	*E. mathaei*	50	3.22 ± 0.16	1.2 ± 0.05	2.67 ± 0.18	Arakaki et al. 1998
	*E*. sp. C	50	3.45 ± 0.16	1.12 ± 0.05	3.07 ± 0.18	Arakaki et al. 1998
	*E. oblonga*	50	5.89 ± 0.31	0.97 ± 0.05	6.11 ± 0.48	Arakaki et al. 1998
Mauritius	EB	20	3.16 ± 0.16	1.27 ± 0.05	2.49 ± 0.15	Arakaki et al. 1998
	VE	20	3.25 ± 0.13	1.34 ± 0.04	2.43 ± 0.12	Arakaki et al. 1998
	BE	20	6.28 ± 0.40	1.06 ± 0.06	5.97 ± 0.55	Arakaki et al. 1998
Gulf of Aqaba/Eilat	EE	150	3.90 ± 0.34	1.27 ± 0.08	3.08 ± 0.29	This study
Bonin	BO	20	6.89 ± 0.45	1.01 ± 0.06	6.34 ± 0.56	Arakaki et al. 1999
Guam	GU	80	2.70 ± 0.17	1.19 ± 0.06	2.27 ± 0.18	Arakaki et al. 1999
Hawaii	HA	42	3.16 ± 0.21	1.19 ± 0.08	2.67 ± 0.26	Arakaki et al. 1999

EE, EB, VE, BE, BO, GU and HA denote Eilat’s *Echinometra*; Mauritian *E. mathaei*, Violet *Echinometra* and *E. oblonga*; Bonin *Echinometra*, Guam’s *Echinometra*, and Hawaiian *Echinometra*, respectively. Figures indicate the mean ± S.D. Sperm were obtained from one individual of each species for Okinawa and Mauritius, five individuals (30 sperm heads from each) for Eilat, and four individuals of each species for Guam and Hawaii.

### Phylogenetic relationship

**Figure 5:**
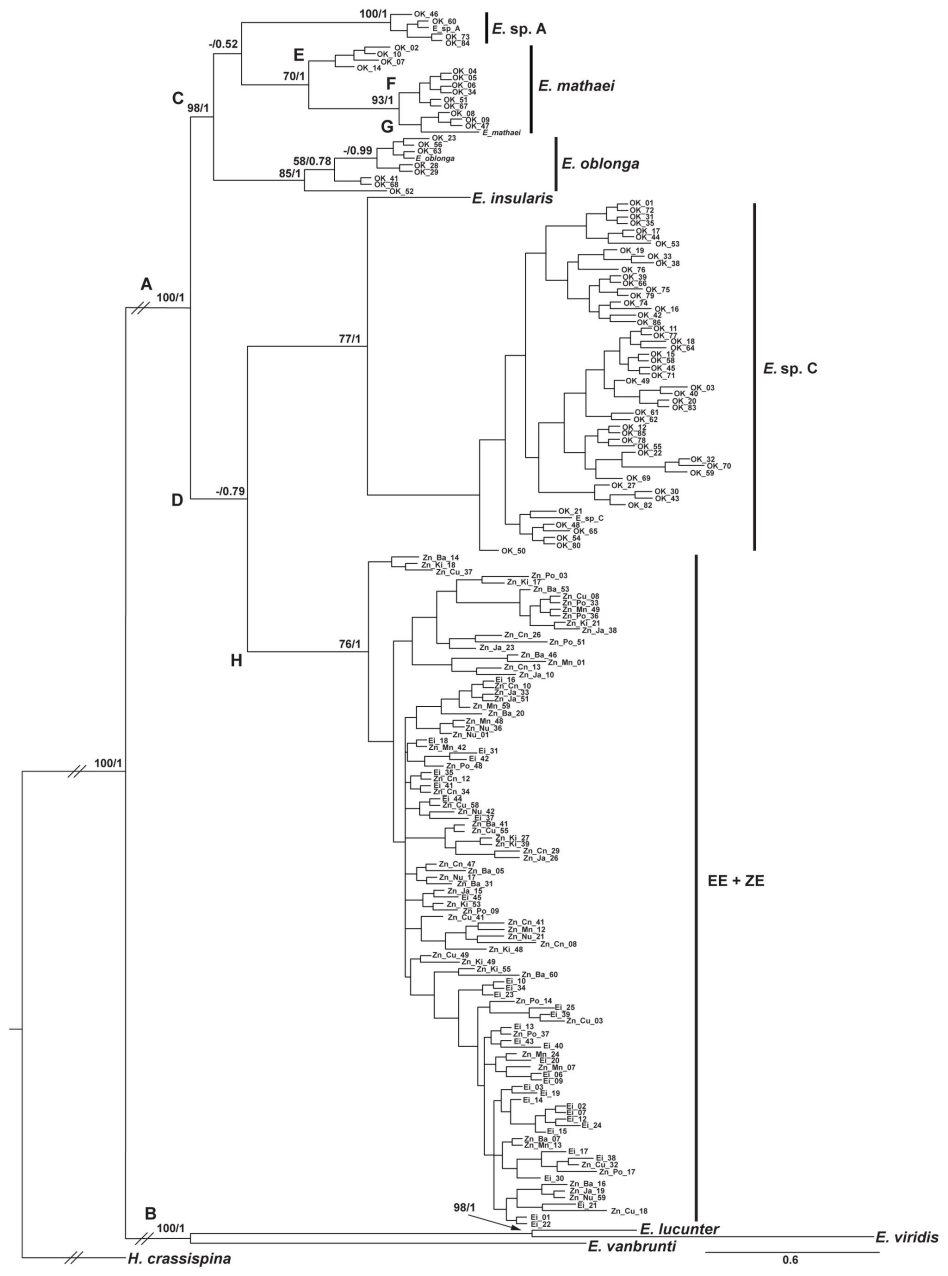
*Echinometra* Bayesian inference phylogenetic reconstruction tree. Consensus tree of 170,000 trees (after burn-in) generated by MCMC analysis of 20 million generations. 544 bp long COI fragments of 189 *Echinometra* specimens were used in the analysis. 181 sequences were generated in the current study corresponding to five *Echinometra* species, and a novel *Echinometra* mitochondrial lineage. Sequences downloaded from GenBank represent the eight known species of *Echinometra* and are denoted *E*. sp. A, *E. mathaei*, *E*. sp. C and *E. oblonga*, *E. insularis*, *E. lucunter*, *E. viridis* and *E. vanbrunti* (accession numbers: AY262861, AY262940, AY262932, AY262886, AY262906; AF255471, AF255530 and AF255526, from Landry et al. [4] and McCartney et al. [7], respectively). *Heliocidaris crassispina* (Echinodermata, Echinoidea) (GenBank accession number: JN716400) was used as outgroup. The supporting > 50% values of 1,000 bootstrap replications of the ML analysis and the posterior probabilities of the BI analysis are shown above nodes respectively. Clades A-H are discussed in the text.

Both Maximum Likelihood (ML) and Bayesian Inference (BI) analyses yielded similar tree topologies and reflected no conflicts with respect to any of the major clades. Therefore, we only present the BI tree (Figure 5). Ingroup taxa were monophyletic (BSP = 100; PP = 1) and clustered into nine main clades. These nine clades correspond to the eight known species of *Echinometra* and a ninth clade that comprises both EE and ZE. These clades clustered partially based on geographic region: clade A comprises all the species from the Indo West Pacific (IWP) and western Indian Ocean (WIO), including the Red Sea and a species from the eastern Pacific (BSP = 100; PP = 1); and clade B comprises western Atlantic and the other eastern Pacific species (BSP = 100; PP = 1). Among the six taxa of clade A, *E*. sp. A, *E. mathaei* and *E. oblonga* together form a group of entirely IWP species (BSP = 100; PP = 1). The other taxa in clade A are comprised of taxa from various geographic regions, including the IWP (*E*. sp. C), eastern Pacific (*E. insularis*), Red Sea (EE) and WIO (ZE) (BSP = 100; PP = 1). The clade containing EE and ZE is sister to the clade containing E. sp. C and *E. insularis*, albeit weakly supported (BSP < 50; PP = 0.79). Intra- and inter-specific Kimura-two-parameter (K2P) genetic divergence among taxa is summarized in Table 5. Intraspecific divergence in EE+ZE (0.70%) was similar to the values observed in *E. mathaei* and *E. oblonga* (0.68% and 0.63%, respectively). These levels of divergence were higher than the intermediate levels present in E. sp. C (0.53%) and more than three-fold that of E. sp. A (0.22%). Interspecific divergence values between EE+ZE and the IWP species showed the greatest distance from E. sp. C (4.39%), followed by *E. oblonga* (3.58%). The distance between *E. mathaei* to E. sp. A (2.90%) was smaller than the distances between EE+ZE and the latter two species (3.03% and 3.19%, respectively). EE+ZE distance from *E. oblonga* (3.58%) was similar to *E. oblonga*’s divergence from E. sp. A (3.59%).

**Table 5:** Intra- and interspecific genetic divergence in *Echinometra*.

	EE & ZE	*E. mathaei*	*E*. sp. A	*E. oblonga*	*E*. sp. C
EE and ZE	**0.70**				
*E. mathaei*	3.03	**0.68**			
*E*. sp. A	3.19	2.90	**0.22**		
*E. oblonga*	3.58	2.90	3.59	**0.63**	
*E*. sp. C	4.39	4.53	5.28	4.20	**0.53**

Intraspecific (diagonal) and interspecific (below diagonal) genetic divergence (%) among WIO and IWP species of *Echinometra* from sequences obtained in the current study. Mitochondrial DNA divergence is based on Kimura two-parameter (K2P) distances at the COI gene.

## Discussion

### Identification based on traditional taxonomy

#### IWP species

The type locality of *Echinometra mathaei* is Mauritius [32]. Consequently, *Echinometra* from adjacent localities such as Zanzibar and even the Red Sea have long been regarded as such [2,8,18,33], despite a lack of sufficient taxonomical background. Color is a prominent external taxonomic feature in many organisms, including sea urchins from the genus *Echinometra* [8]. However, it is clear that spine color alone is insufficient to determine species affiliation in most Okinawan *Echinometra*, since all but one species share spine colors. The exception is E. sp. A, in which a single color morph was observed. The high plasticity of spine color and its subjective terminology thus limit the effectiveness of this character as a delineating feature in *Echinometra*. Another character prone to ambiguity is the skin around the peristome. This character is as variable in color as the spines themselves. However, unlike the spines in which seven distinct character-states are used to define their variety, in the skin around the peristome, only two are used. When applied to a plastic feature such as the color of this skin, this may lead to conflicting decisions. Milled rings are also only partially informative. In E. sp. A and *E. oblonga* all individuals presented a single character-state (Table 2). While findings from these two species correspond well with the literature [3], in E. sp. C and *E. mathaei* great differences were found. Arakaki et al. [3] describes E. sp. C and *E. mathaei* as having completely dark and completely bright milled rings, respectively, making this character useful in delineating Okinawan species. However, in this study we found that both species feature bright and dark rings simultaneously (Table 2). The reason for these differences may be attributed to the low number of samples obtained by Arakaki et al. (a total of n = 21 for both species), in comparison to the 72 samples collected in the current study. In contrast to coloration, spicules of the tube feet and gonads were clearly distinguishable and unambiguous. Here too sample size was relevant: in the current study, the 45 *E. mathaei* and 27 E. sp. C sampled yielded five and four tube feet spicules character-states, respectively; whereas Arakaki et al. [3] found only two tube feet spicule character-states in *E. mathaei* and three in E. sp. C, for their substantially lower sample sizes of 11 and 10, respectively. Clearly, a larger number of samples are more likely to represent the proportion of natural states, and offer a better prospective for identifying subtle morphological differences in closely-related species, such as IWP *Echinometra*. Nonetheless, even with thorough sampling *Echinometra* may present a high plasticity of morphological characters. In contrast to previous studies [34], we found lower proportions of bihamate spicules and higher proportions of triradiate spicules (29% and 38%, vs. 69%-85% and 15%-31%, respectively) in the tube feet of *E. mathaei*. Moreover, while Rahman and Uehara [34] report no bihamate gonad spicules in *E. oblonga* from Okinawa, we found these spicules in 20% of the population from the same location. Hence, large intraspecific variations and inconsistencies in morphology and even reproduction [34], are present in IWP *Echinometra*.

#### WIO and Red Sea species

ZE and EE appear to be the same species based on their external morphological features, as they share some unique morphological features and express a high resemblance in others. For example, a unique character-state only found in populations from these two locations is the ‘faded’ milled rings, which are not apparent in any of the Okinawan urchins. In other characters too, ZE and EE show highly similar proportions of character-states. One example of such similarity is seen in the character ‘skin of peristome’, in which both populations present, in highly similar proportions, predominantly ‘dark’-skinned individuals with only a few ‘bright’-skinned ones (Table 2).

The spine coloration of urchins from these two localities may provide some explanation as to why these populations were mistaken for *E. mathaei*. Although ZE are generally darker than EE (Table 2), urchins from both localities also share the light brown color morph, similar to *E. mathaei*. Moreover, ZE and EE presented the ‘bihamate’ spicule type in the tube feet in all of the samples studied. *E. mathaei*, though also presenting this spicule type, did so in less than 30% of its population. In the gonads of both EE and ZE, 'needle' type spicules were always present, either solely (70% and 60.8% of samples, respectively) or in combinations with other spicule types. These proportions were twice as high as in *E. mathaei* (only 35.5%). Furthermore, in almost 60% of *E. matheai*’s population the needle spicule was entirely absent. Finally, in the gonads of both EE and ZE, the ‘figure-eight’ shaped spicules were eminent in 20% and 27.45% of the samples, respectively, in contrast to the other *Echinometra* species were these spicules where nearly absent. In fact, the only time these spicules were observed in a species other than EE or ZE, was in E. sp. C, and only in the ‘multiple’ spicule combination that occurred in no more than 3.7% of the samples taken. The differences in spicule types and arrangements between ZE and EE, and the rest of the IWP *Echinometra*, together with the stability of spicules as a morphological marker, suggest that urchins from the two former populations may differ from the IWP species. Though originally oversimplified: "Four pore pairs to the arc is the normal number in *Echinometra mathaei*, whereas the other species of *Echinometra* have a greater number, 5-8" [8], this character was considered stable even in more recent detailed *Echinometra* studies [3]. However, the number of pore-pairs was limited in its ability to differentiate species, as some species shared similar pore pair distributions (Table 3). In fact, among the four IWP species, significant differences in pore pair distribution were only found between *E. oblonga* and *E. mathaei*, while the rest of these species had indiscriminate distributions (Table 3). Other results from this analysis, which might otherwise be informative, such as the apparent difference between EE and ZE, or ZE’s difference from both Okinawan and Mauritian *E. mathaei*, must therefore be doubted. Our findings thus question the use of this character as a useful feature for discriminating among the species of *Echinometra*.

Sperm size in sea urchins can be polymorphic among populations within species [3]; however, a large variation in length (i.e. more than two-fold) usually represents differences in species [4,35–37]. The primary difference lies in the tip of the sperm head and the shape of the nucleus [3]. In the current study, the sperm morphology of EE resembled that of two other *Echinometra* species described in the literature, *E. mathaei* and *E*. sp. C. That no significant difference in sperm morphology was found between these species (Table 4) implies a certain degree of relatedness between them. Although *E. oblonga* type locality was never described [32] its presence in the WIO was reported several times in the scientific literature, including some populations in Mauritius [3,38]. However, considerable differences in sperm morphology (Figure 4, Table 4), tube feet and gonad spicules, reject the assumption that EE and ZE could in fact be *E. oblonga*.

Since spicule assembly, the number of pore-pairs, and the morphology of the sperm in the Okinawan *Echinometra* species complex reflect species level differences [3,6], it is possible that *Echinometra* from Eilat and Zanzibar should be regarded as a new species.

### Molecular taxonomy and phylogenetic relationships

Both ML and BI methods produced tree topologies that reaffirm the phylogenetic relationships among the eight known *Echinometra* species, with the results from the current study reflecting *Echinometra* phylogenies published elsewhere [13,39]. To truly represent *Echinometra* diversity at the sites we studied, and in order to avoid bias due to small sample size [28,29], we aimed at acquiring a large data set from each of the study sites. Thus, our data facilitates the detection of intraspecific variation of each of the species studied. In *E. mathaei*, for example, three well-defined sub-clades could be distinguished. These sub-clades (referred to as E, F and G; Figure 5), suggest the recent divergence of IWP *E. mathaei* into two or perhaps even three mitochondrial lineages and possibly new species. However, genetic divergence of these sub-clades was low (0.2%-1.4%), well within the intraspecific divergence boundaries of *Echinometra*. Nonetheless, as the current analysis is based on a single mitochondrial gene, incorporation of nuclear genes are thus needed to check if interbreeding still exists or not between these mitochondrial lineages.

The deepest split of IWP *Echinometra* occurred between E. sp. C and its sister species, the endemic Easter Island *E. insularis*, and the cluster of E. sp. A, *E. mathaei* and *E. oblonga* (cluster C). These findings are supported by previous phylogenetic studies of *Echinometra* [4,13,39]. *E. mathaei* is similarly distant from E. sp. A as it is from *E. oblonga* (2.90%), while the latter two are further diverged from one another (3.59%). These genetic distances are identical to those suggested by Palumbi [40], and make *E. mathaei* and *E*. sp. A the most closely-related pair of *Echinometra* species. Moreover, our findings support the claim of sister-species relationship between *E. mathaei* and *E*. sp. A, as suggested by Landry et al. 2003. The poor bootstrap support for the branching of these three species (i.e. *E. mathaei*, *E*. sp. A, and *E. oblonga*) thus implies that their genetic structure is not completely resolved and requires further clarification, in particular by adding data from nuclear genes.

The phylogeny of *Echinometra* from Eilat and Zanzibar is presented here for the first time (Figure 5). EE and ZE form a monophyletic clade (clade H) that branched off from the cluster of E. sp. C and *E. insularis* (clade D). Degrees of divergence of clade H from the rest of *Echinometra* (3.03%-4.39%) are well within the interspecific range for this genus [39], and are higher than the distance between many of the other IWP species. Thus mtDNA data suggest that *Echinometra* from the Red Sea and WIO differ from the other *Echinometra* species. That no clear pattern of genetic structure, neither between nor within the latter two populations was found, despite our thorough sampling at these sites, suggests they are comprised of the same species. Whether a single or multiple species exist in the entire WIO and Red Sea regions nonetheless needs further clarification, as our sampling was limited to only two locations (i.e. Eilat in the Red Sea and Zanzibar in the WIO). The numerous possible habitats and the vastness of the Indian Ocean hold great prospects for identifying other *Echinometra* species in that region. Expanding our search to new sites, utilizing both meticulous morphological analysis combined with molecular evidence, will deepen our understanding of *Echinometra* diversity and distribution and of marine speciation processes as a whole.

## Conclusions

The morphological keys available for identifying species of the sea urchin *Echinometra* were tested on four closely-related Okinawan *Echinometra* and were generally in agreement with the molecular taxonomy. However, an in-depth examination of the characters used for identification of these species revealed a much higher intraspecific diversity than previously reported. The COI phylogenetic reconstruction of Okinawan *Echinometra* strongly reconfirmed their current ascription as four distinct species. While *Echinometra* from Eilat and Zanzibar had been previously mistaken for *E. mathaei*, current data suggest that *Echinometra* from the latter two regions may in fact be a new, undescribed, species. Yet more work is needed to elucidate the long list of synonyms currently subsumed in *Echinometra*. Thus, an integrated approach that combines both traditional and molecular taxonomic methodologies should be applied in order to satisfy the intricate demands of assigning correct scientific names to taxa.

## References

[1] Motokawa T (1991) Introduction to the symposium Echinometra a complex under speciation. In: Yanagisawa T Yasumasu I Oguro C Suzuki N Motokawa T Biology of Echinodermata. Rotterdam: Balkema Press p. 89.

[2] McClanahan T, Muthiga N (2001) Ecology of Echinometra. In: Lawrence M Edible Sea Urchins: Biology and Ecology. 2 ed. Amsterdam: Elsevier pp. 297-317.

[3] Arakaki Y, Uehara T, Fagoonee I (1998) Comparative Studies of the Genus Echinometra from Okinawa and Mauritius. Zool Sci 15: 159-168. doi:10.2108/zsj.15.159. PubMed: 18429667. 18429667

[4] Landry C, Geyer LB, Arakaki Y, Uehara T, Palumbi SR (2003) Recent speciation in the Indo-West Pacific: rapid evolution of gamete recognition and sperm morphology in cryptic species of sea urchin. Proc R Soc Lond B 270: 1839-1847. doi:10.1098/rspb.2003.2395. PubMed: 12964987. PMC1691439 12964987

[5] Arakaki Y, Uehara T (1999) Morphological comparison of black Echinometra individuals among those in the Indo-West Pacific. Zool Sci 16: 551-558. doi:10.2108/zsj.16.551.

[6] Palumbi SR, Metz EC (1991) Strong reproductive isolation between closely related tropical sea urchins (genus Echinometra). Mol Biol Evol 8: 227-239. PubMed: 2046543. 2046543 10.1093/oxfordjournals.molbev.a040642

[7] McCartney MA, Keller G, Lessios HA (2000) Dispersal barriers in tropical oceans and speciation in Atlantic and eastern Pacific sea urchins of the genus Echinometra. Mol Ecol 9: 1391-1400. doi:10.1046/j.1365-294x.2000.01022.x. PubMed: 10972777. 10972777

[8] Mortensen T (1943) A monograph of the Echinoidea: Camarondonta. In: Reitzel CA Denmark: Copenhagen.

[9] Mayr E (1954) Geographic Speciation in Tropical Echinoids. Evolution 8: 1-18. doi:10.2307/2405661.

[10] Berlocher SH (1998) Can sympatric speciation be proven from phylogenetic and biogeographic evidence? In: Howard DJ Berlocher SH Endless Forms: Species and Speciation. Oxford University Press pp. 99-113.

[11] Matsuoka N, Hatanaka T (1991) Molecular Evidence for the Existence of Four Sibling Species within the Sea-Urchin, Echinometra mathaei in Japanese Waters and their Evolutionary Relationships. Zool Sci 8: 121-133.

[12] Metz EC, Palumbi SR (1996) Positive Selection and Sequence Rearrangements Generate Extensive Polymorphism in the Gamete Recognition Protein Bindin. Mol Biol Evol 13: 397-406. doi:10.1093/oxfordjournals.molbev.a025598. PubMed: 8587504. 8587504

[13] Palumbi SR (1996) What can molecular genetics contribute to marine biogeography? An urchin’s tale. J Exp Mar Biol Ecol 203: 75-92. doi:10.1016/0022-0981(96)02571-3.

[14] Palumbi SR, Grabowsky G, Duda T, Geyer L, Tachino N (1997) Speciation and population genetic structure in tropical Pacific sea urchins. Evolution 51: 1506-1517. doi:10.2307/2411203. 28568622

[15] Kelso DP (1970) A comparative morphological and ecological Study of two species of the sea urchin genus Echinometra in Hawaii. Honolulu: University of Hawaii . p. 112

[16] Mayr E (1942) Systematics and the origin of species, from the viewpoint of a zoologist. Cambridge, MA: Harvard University Press.

[17] Russo AR (1977) Water flow and the distribution and abundance of echinoids (genus: *Echinometra*) on an Hawaiian Reef. Mar Freshw Res 28: 693-702. doi:10.1071/MF9770693.

[18] Lawrence JM (1983) Alternate states of populations of *Echinometra* *mathaei* (De Blainville) (Echinodermata: echinoidea) in the Gulf of Suez and the Gulf of Aqaba. Bulletin Institute Oceanography Fisheries 9: 141-147.

[19] Clark AM, Rowe FWE (1971) Monograph of shallow-water Indo-west Pacific echinoderms. Trustees of the British Museum (Natural History) 690

[20] Bermingham E, Lessios HA (1993) Rate variation of protein and mitochondrial DNA evolution as revealed by sea urchins separated by the Isthmus of Panama. Proc Natl Acad Sci U S A 90: 2734-2738. doi:10.1073/pnas.90.7.2734. PubMed: 8096641. 8096641 PMC46170

[21] Lessios HA, Kessing BD, Robertson DR (1998) Massive Gene Flow across the World’s Most Potent Marine Biogeographic Barrier. Proc R Soc Lond B Biol Sci 265: 583-588. doi:10.1098/rspb.1998.0334.

[22] Abràmoff MD, Magalhães PJ, Ram SJ (2004) Image processing with ImageJ. Biophotonics Int 11: 36-42.

[23] Pochon X, Pawlowski J, Zaninetti L, Rowan R (2001) High genetic diversity and relative specificity among Symbiodinium- like endosymbiotic dinoflagellates of sortid foraminiferans. Mar Biol 139: 1069-1078. doi:10.1007/s002270100674.

[24] Geyer LB, Palumbi SR (2003) Reproductive character displacement and the genetics of gamete recognition in tropical urchins. Evolution 57: 1049-1060. doi:10.1111/j.0014-3820.2003.tb00315.x. PubMed: 12836822. 12836822

[25] Katoh K, Toh H (2010) Parallelization of the MAFFT multiple sequence alignment program. Bioinformatics 26: 1899-1900. doi:10.1093/bioinformatics/btq224. PubMed: 20427515. 20427515 PMC2905546

[26] Silvestro D, Michalak I (2011) raxmlGUI: a graphical front-end for RAxML. Organ Divers Evol 12: 335-337.

[27] Ronquist F, Huelsenbeck JP (2003) MrBayes 3: Bayesian phylogenetic inference under mixed models. Bioinformatics 19: 1572-1574. doi:10.1093/bioinformatics/btg180. PubMed: 12912839. 12912839

[28] Funk DJ, Omland KE (2003) Species-level paraphyly and polyphyly: frequency, causes, and consequences, with Insights from animal mitochondrial DNA. Annu Rev Ecol Evol Syst 34: 397-423. doi:10.1146/annurev.ecolsys.34.011802.132421.

[29] Meyer CP, Paulay G (2005) DNA barcoding: error rates based on comprehensive sampling. PLOS Biol 3: e422. doi:10.1371/journal.pbio.0030422. PubMed: 16336051. 16336051 PMC1287506

[30] Kimura M (1980) A simple method for estimating evolutionary rates of base substitutions through comparative studies of nucleotide sequences. J Mol Evol 16: 111-120. doi:10.1007/BF01731581. PubMed: 7463489. 7463489

[31] Tamura K, Peterson D, Peterson N, Stecher G, Nei M et al. (2011) MEGA5: Molecular Evolutionary Genetics Analysis Using Maximum Likelihood, Evolutionary Distance, and Maximum Parsimony Methods. Mol Biol Evol 28: 2731-2739. doi:10.1093/molbev/msr121. PubMed: 21546353. 21546353 PMC3203626

[32] Blainville H (1825) Dictionnaire des sciences naturelles, dans lequel un traité méthodiquement des différences tetres de la nature.

[33] Fishelson L (1971) Ecology and distribution of the benthic fauna in the shallow waters of the Red Sea. Mar Biol 10: 113-133. doi:10.1007/BF00354828.

[34] Rahman SM, Uehara T (2004) Interspecific and intraspecific variations in sibling species of sea urchin Echinometra. Comp Biochem Physiol A Mol Integr Physiol 139: 469-478. doi:10.1016/j.cbpb.2004.10.005. PubMed: 15596392. 15596392

[35] Chia FS, Atwood D, Crawford B (1975) Comparative morphology of echinoderm sperm and possible phylogenetic implications. Am Zool 15: 553-565.

[36] Amy RL (1983) Gamete sizes and developmental timetables of five tropical sea urchins. Bull Mar Sci 33: 173-176.

[37] Raff RA, Herlands L, Morris VB, Healy J (1990) Evolutionary modification of echinoid sperm correlates with developmental mode. Dev Growth Differ 32: 283-291. doi:10.1111/j.1440-169X.1990.00283.x. 37281013

[38] Michel C (1974) Notes on marine biology studies made in Mauritius. The Mauritius Institute. 284pp.

[39] Palumbi SR, Lessios HA (2005) Evolutionary animation: How do molecular phylogenies compare to Mayr’s reconstruction of speciation patterns in the sea? Proc Natl Acad Sci USA 102: 6566-6572. doi:10.1073/pnas.0501806102. PubMed: 15851681. 15851681 PMC1131860

[40] Palumbi SR (1997) Molecular biogeography of the Pacific. Coral Reefs 16: S47-S52. doi:10.1007/s003380050058.

